# Biomarker Research in Parkinson’s Disease Using Metabolite Profiling

**DOI:** 10.3390/metabo7030042

**Published:** 2017-08-11

**Authors:** Jesper F. Havelund, Niels H. H. Heegaard, Nils J. K. Færgeman, Jan Bert Gramsbergen

**Affiliations:** 1 Villum Centre for Bioanalytical Sciences, Department of Biochemistry and Molecular Biology, University of Southern Denmark, DK-5230 Odense, Denmark; jhav@bmb.sdu.dk (J.F.H.); nils.f@bmb.sdu.dk (N.J.K.F.); 2 Department of Autoimmunology and Biomarkers, Statens Serum Institute, DK-2300 Copenhagen, Denmark; nhe@ssi.dk; 3 Department of Clinical Biochemistry and Pharmacology, Odense University Hospital, University of Southern Denmark, DK-5000 Odense, Denmark; 4 Institute of Molecular Medicine, University of Southern Denmark, DK-5000 Odense, Denmark

**Keywords:** biomarker, metabolite profiling, metabolomics, Parkinson’s disease

## Abstract

Biomarker research in Parkinson’s disease (PD) has long been dominated by measuring dopamine metabolites or alpha-synuclein in cerebrospinal fluid. However, these markers do not allow early detection, precise prognosis or monitoring of disease progression. Moreover, PD is now considered a multifactorial disease, which requires a more precise diagnosis and personalized medication to obtain optimal outcome. In recent years, advanced metabolite profiling of body fluids like serum/plasma, CSF or urine, known as “metabolomics”, has become a powerful and promising tool to identify novel biomarkers or “metabolic fingerprints” characteristic for PD at various stages of disease. In this review, we discuss metabolite profiling in clinical and experimental PD. We briefly review the use of different analytical platforms and methodologies and discuss the obtained results, the involved metabolic pathways, the potential as a biomarker and the significance of understanding the pathophysiology of PD. Many of the studies report alterations in alanine, branched-chain amino acids and fatty acid metabolism, all pointing to mitochondrial dysfunction in PD. Aromatic amino acids (phenylalanine, tyrosine, tryptophan) and purine metabolism (uric acid) are also altered in most metabolite profiling studies in PD.

## 1. Introduction

Parkinson’s disease (PD) is a common, age-related progressive neurodegenerative disease, affecting more than 1% of the population over 60 years of age [1]. Diagnosis of PD relies on clinical history, physical examination and the response to dopaminergic drugs, but misdiagnosis is common in early phases of the disease [2]. Clinical symptoms, such as hypokinesia, tremor at rest, rigidity and/or postural instability first appear after patients have lost about 80% of dopaminergic nerve terminals in the striatum as assessed in vivo using SPECT or PET imaging for the dopamine transporter (DAT) [3,4] or lost 30–50% of dopaminergic cell bodies in substantia nigra as assessed post-mortem [5]. In the prodromal phase of PD where motor symptoms are not yet apparent patients may suffer from hyposmia, rapid eye movement (REM) sleep behavior disorder, depression and/or constipation [6,7]. Dopaminergic changes observed on DAT scans appear relatively late in the disease process. Moreover, DAT scans cannot differentiate PD from multi-system atrophy (MSA), progressive supranuclear palsy (PSP) or other neurological or neurotoxic disorders associated with severe loss of dopaminergic nigrostriatal terminals. The final confirmation of the PD diagnosis depends on post-mortem examination of brain tissue for the presence of alpha-synuclein-containing Lewy bodies and the loss of nigral neurons.

Despite extensive preclinical research in animal models of PD, there are still no effective neuroprotective or disease-modifying drugs for PD on the market. Thus, current drug treatment for PD continues to be based on symptomatic, dopamine replacement therapies, in some cases supplemented with antidyskinetic drugs, such as amantadine [8]. After several years of treatment with L-DOPA and related drugs, the majority of PD patients develop L-DOPA-induced-dyskinesia (LID), which is a serious, irreversible adverse reaction to chronic treatment in more advanced stages of PD [9]. At present, it is not possible to predict which PD patients are at risk of developing LID.

PD is a multifactorial disorder where genetic factors, environmental exposures and aging contribute to the risk of developing the disease. There is a large variability in the onset and course of the disease. In both sporadic and genetic PD, the underlying pathophysiological and pathogenetic mechanisms include mitochondrial dysfunction, oxidative stress, Lewy body pathology (alpha-synuclein aggregation) and deficits in proteasomal function or autophagy-lysosomal degradation of defective proteins (e.g., alpha-synuclein) [10,11,12,13]. The majority of PD cases have no known monogenetic etiology, but a subset of cases is associated with mutations in alpha-synuclein, LRRK2, Parkin, PINK1, DJ-1 [14,15] or glucocerebrosidase (GBA) and other lysosomal enzymes [16,17,18,19,20].

After the discovery of the key roles of alpha-synuclein in Lewy body pathology in PD and related disorders (MSA, PSP) and tau, phosphorylated-tau and amyloid-beta (1-42) in neurofibrillary tangles and plaques in Alzheimer’s disease (AD), major research efforts have been directed to the measurements of these proteins in body fluids, particularly in CSF. In the Parkinson’s Progression Markers Initiative study, these markers were measured in a large cohort study of patients with newly-diagnosed PD, but the diagnostic and prognostic value of these markers was limited due to large overlap between healthy controls and early PD [21]. In this study, the level of alpha-synuclein was lower in PD patients with the non-tremor dominant phenotype, compared with the tremor-dominant phenotype. Another longitudinal study of CSF alpha-synuclein observed increases of alpha-synuclein over two years in patients with a long disease duration, but not in patients with a short disease duration [22], further complicating the use of alpha-synuclein as a diagnostic and prognostic marker. Changes in CSF amyloid-beta (1-42) and tau have been related to cognitive decline in PD, but only in combination with information on age of onset, non-motor assessments and DAT imaging do these CSF markers have a predictive value [23].

Although much progress has been made in PD biomarker research as illustrated by the work cited above, clinically useful biochemical markers remain to be identified and validated for early and more precise diagnosis of PD or for differentiation of subtypes of PD, which may require different treatments. Biomarkers are also needed to predict the course of the disease including possible adverse effects of dopamine replacement strategies, the development of LID and to monitor the effect of experimental disease-modifying treatments in the future. Since PD is now considered to be a diverse group of disorders, rather than a single pathogenic disease entity, affecting both the peripheral and central nervous system [7,24], it has been suggested that precision medicine applied to PD should be based on biomarker profiles instead of clinical features, which can change rapidly within a few years and which overlap to a large extent [25].

## 2. Metabolites as Biomarkers

Before the discovery of genetic forms of PD and the development of sensitive assays to detect proteins associated with PD pathology in body fluids, biomarker studies for PD focused on changes in small molecules/metabolites (mainly in CSF), such as catecholamines, serotonin, amino acids (including neurotransmitters like GABA, glycine, glutamate or precursors of the monoamine neurotransmitters including phenylalanine, tyrosine, tryptophan and related compounds) using HPLC with electrochemical, fluorescent or UV detection. Other small molecules of interest are glutathione and purine metabolites, including uric acid, because of their role as antioxidants [26,27,28].

For original articles on catecholamines and metabolites as biomarkers in PD, we refer to [29,30,31] and for recent reviews [32,33,34]. Loss of catecholamines and their metabolites in PD biofluids can only reliably be detected in drug-naive patients or after long-term drug (L-DOPA) wash-out. Furthermore, CSF levels of the dopamine (DA) metabolite homovanillic acid (HVA) appear to be a less reliable marker for loss of central DA than CSF levels of the other DA metabolite, dihydroxyphenylacetic acid (DOPAC), which shows high accuracy in separating PD patients, including recently-diagnosed patients, from controls [29,30,31].

Changes in CSF amino acids assessed by conventional HPLC techniques have been extensively reviewed [34,35]. The various results obtained for CSF amino acids levels in PD do not allow firm conclusions regarding which pathways are affected in PD. The lack of consistent results may be due to the lack of adequate matching of cases and controls, differences in anti-Parkinson therapy, which is a major confounding factor, and differences in storage and processing of samples. Amino acid metabolism in PD has also been studied by more recent metabolomic approaches using nuclear magnetic resonance (NMR) and liquid chromatography-mass spectrometry (LC-MS). Results obtained in clinical studies of body fluids are summarized in Table 1 and results of experimental studies in Table 2.

Proton (^1^H) and phosphorus (^31^P) magnetic resonance spectroscopy (MRS) are noninvasive imaging techniques that have been used to the study metabolites involved in energy metabolism (e.g., lactate, creatine, phosphocreatine, ATP) and other abundant metabolites (e.g., *N*-acetylaspartate, a neuronal marker or choline-containing compounds) in vivo in brain tissues. Some ^1^HMRS studies reported elevations of striatal lactate in PD or animal models of PD [36,37], but most studies report a reduction of *N*-acetylaspartate/creatine or *N*-acetylaspartate/choline ratios in advanced, but not in early PD [38,39,40,41].

In the next sections, we discuss the methodology and analytical platforms used in metabolomic studies and summarize the major findings of metabolite profiling studies in PD.

## 3. Metabolomics in PD

In diabetes and cancer research, metabolic profiling has played a major role in uncovering specific metabolic pathways associated with disease processes and characterizing subtypes of, e.g., prostate or breast cancer [42]. We are not yet at this stage in PD metabolite profiling research. The aim of metabolic studies in PD is to identify candidate biomarkers or, more likely, provide a metabolic fingerprint for early diagnosis and prognosis, monitoring disease progression and the effect of disease-modifying treatments, as well as to gain a better understanding of the molecular pathogenesis of PD or its various genetic and/or sporadic subtypes. Metabolic profiling of tissues or biofluids reflects the complex interaction of genes, proteins and the internal and external environment of an individual. Differences between metabolic profiles of individuals or groups of healthy or diseased persons can be the result of differences in genetic background, epigenetic modifications, lifestyle, stress, pathogen exposure, diet, medication, gut flora, and so on. The first metabolomic studies in neurodegenerative diseases, including PD and Alzheimer’s disease (AD) have been reviewed by various authors [43,44,45,46].

### 3.1. Analytical Challenges to Assess the “Metabolome”

Recent developments in analytical chemistry, especially liquid chromatography-mass spectrometry (LC-MS) and gas chromatography-mass spectrometry (GC-MS) and the availability of extensive software tools for analyzing big data allow the detection, identification and quantification of very large numbers of molecules in the micro- and nano-molar range in body fluids, such as CSF, blood and urine. Metabolomics refers to the study of the entire complement of metabolites (or small molecules) in a biological system: the “metabolome” [47]. The genome, transcriptome and proteome, where the basic analyte molecular structure is made up by polymers based on four nucleotides or 20 encoded and additional derived amino acids can be subjected to in-depth analysis using a single instrumental setup, respectively. The metabolome, however, is much more chemically diverse, and thus, multiple approaches involving different extraction and analytical methods must be applied to obtain unbiased and complete information about the entire metabolome (see the reviews on this topic [48,49]).

### 3.2. Sample Preparation, Extraction Procedures and Analytical Platforms

The extraction methods in metabolomics-driven PD research (regardless of sample type) consist primarily of protein removal by precipitation in organic solvent and centrifugation (e.g., [50,51,52]) or by centrifugal filtration prior to lyophilization. Samples for GC-MS are derivatized to increase volatility and thermal stability before sample injection and analysis [50,53,54]. Samples for LC-MS, NMR or the liquid chromatography electrochemistry array (LCECA) are typically resuspended in a small volume of analysis-friendly solvent (containing deuterium for NMR) before injection (e.g., [51,55,56]).

The analytical platforms can be divided into at least four groups: LCECA, NMR, GC-MS and LC-MS, which have been excellently reviewed [49,57,58]. In brief, LCECA contains multiple coulometric electrodes in an array allowing detection and quantification of compounds as a function of their retention times and oxidation-reduction potentials [59,60]. This method has very high sensitivity and reproducibility, but relatively low throughput due to longer run times (e.g., 110 min [51,56]) of consecutive samples and yields no structural information. NMR is based on atom-centered interactions and can provide high level structural information (reviewed in [61]). In GC-MS, the compounds are typically derivatized and then separated in the gas-phase on a long column before mass spectrometric analysis (mass over charge separation). In LC-MS, the analytes are separated in solution and ionized before analysis in the MS (see [62] for an excellent book on mass spectrometry). NMR, GC-MS and LC-MS are frequently used for metabolomics (see Table 1) as they provide structural information, have high throughput and can analyze a large variety and mixtures of compounds. The main advantage of NMR is the ability to identify novel compounds where the MS-based approaches need reference information in order to identify a compound. On the other hand, the sensitivity of NMR is several orders of magnitude lower than the sensitivity of GC-MS and LC-MS [49,63]. Compounds in the nM range cannot be detected by NMR, as too large volumes would be needed, and thus, the MS-based approaches are preferred to detect and quantify compounds in this nM range, which is also apparent from Table 1. However, pre-analytical enrichment procedures can be needed also for LC-MS (or LCECA) to measure compounds in the low nM range (e.g., catecholamines in plasma or CSF) [31,64]. NMR can detect water-soluble protonable species, whereas LC-MS and GC-MS provide a much broader profile of compounds, which, however, can only be identified with access to reference information. GC-MS is most suitable for less polar and volatile compounds, whereas LC-MS allows detection of an even broader profile of metabolites and is generally preferred for the more polar compounds, all depending on the setup (discussed in [58,65]). To get a profile as broad as possible, the samples are often analyzed with both GC-MS and LC-MS, where the latter is used in both negative and positive ion mode, which favors acidic and basic compounds, respectively (e.g., [53,66,67]).

The analytical platforms needed for comprehensive metabolite profiling are quite expensive, and most laboratories cannot afford to acquire them all. Thus, research groups should choose the most appropriate instrument based on the chemical characteristics and abundance of the compounds of interest.

### 3.3. Identification and Quantification of Small Molecules

When no synthetic reference standard is available, the identification of compounds is a major challenge in non-NMR-based metabolomics. Reports on the level of confidence in the identification of compounds have been inconsistent, and thus, The Metabolomics Standards Initiative (MSI) was launched 10 years ago, and more recently, the Coordination of Standards in Metabolomics (COSMOS) was initiated [68]. Summer et al. [69] reported that confident identification should be based on a minimum of two pieces of information related to a reference standard (e.g., retention time, fragment ions and accurate mass), whereas less confident identification should only be referred to as putatively-annotated compounds or unknowns. In untargeted MS-based metabolomics, a large part of the extracted compounds comprises unknowns or only putatively identified ones (e.g., [70,71,72]). Challenges in the identification of small molecules using LC-MS have been described elsewhere [63,73].

To allow quantitative comparison with the literature (e.g., to compare control groups in different studies) or to compare the quantity of different compounds, absolute quantification is required. This requires inclusion of standards of known amounts and is thus most commonly only used in targeted metabolomics. External standards are used in LCECA, whereas internal isotope-labelled standards are optimal for absolute quantification in MS and NMR. In MS, the signal intensity is not only dependent on the ion amount, but also very much on the chemical structure of the analytes and on the sample matrix (potentially causing signal suppression). Thus, an isotope-labelled standard is required for absolute quantification of the individual compound as has been demonstrated for a few compounds in Havelund et al. [52]. Metabolite quantification has been reviewed for NMR [74] and for MS-based metabolomics [75].

### 3.4. Untargeted versus Targeted Analysis at Acquisition or Data Analysis Levels

The mass spectrometry-based analysis in the PD-related studies reviewed here (Table 1) is performed as untargeted at the analytical level, without pre-selection of compounds for fragmentation. Some of the LC-MS-based approaches use MS/MS with data-dependent acquisition (DDA), only fragmenting the most intense ions in a spectrum [53,66,71], whereas others run the samples again after the statistical analysis in a targeted analytical setup only focusing on the identification of the statistically interesting compounds [67,70,72,76]. In the GC-MS studies, MS/MS is not utilized, as fragmentation already is induced by the use of electron ionization (EI). Not only the data acquisition, but also the data analysis may be regarded as targeted or untargeted. In the latter case, all compounds (typically more than 500) are extracted and aligned automatically using different specific settings and thresholds. Some low abundant or bad peak shape compounds might thus not be extracted in the subsequent statistical analysis. In the targeted analysis, however, only a subset of compounds (e.g., the identified analytes) is used in the data analysis, allowing manual inspection of all peaks and correction of miss- or non-assigned peaks. Targeted and untargeted metabolomics have been reviewed in [77].

### 3.5. Statistical Analysis

Multivariate statistics such as principal component analysis (PCA) or partial least squares discriminant analysis (PLS-DA) are often used to generate metabolic fingerprints or to find the compounds most responsible for group separation, especially in untargeted (or large-scale targeted) metabolomics [50,51,54,71,76]. Hereby, the relative differences in the metabolomes of different groups (e.g., PD versus controls or different PD subgroups) are determined. In brief, PCA is unsupervised, only showing group separation when within-group variation is sufficiently less than between-group variation and, thus, often fails to separate the groups of interest [50,71]. The supervised approaches like PLS-DA rely on class membership of each observation and overfit models to the data, which most often results in a clear group separation. However, care must be taken as group separation can be achieved with random data [78]. Therefore, results from supervised statistics must always be validated [79] and combined with univariate statistics (references from Table 1). Validation is often lacking in PD-based metabolomic research [50,51,54], possibly contributing to the high degree of variation of PD metabolomics data. Multivariate statistics in metabolomics have been excellently reviewed elsewhere [78]. The performance of a biomarker in terms of specificity and sensitivity can be evaluated using receiver operator characteristic (ROC) curves (reviewed in [80] and applied in [53,67]), a method widely considered the most objective and valid for this purpose [81,82,83,84].

## 4. Metabolomics Studies in PD Patients and Experimental Models

### 4.1. Metabolomic Studies in PD Patients (Table 1)

In Table 1, we have summarized the main findings of metabolite profiling studies in PD over the last decade. We have found only 15 clinical studies using “metabolomics” or “metabolite profiling” to identify biomarkers for PD. It has been suggested that metabolomics studies should include at least 20 patients per group (disease versus control) [85], which is the case for most of the studies in Table 1. However, in the studies of [52,66], less controls were available, and comparison of subgroups of PD (e.g., LRRK2 mutations) in [51,52] (patients with and without LID) resulted also in lower numbers per group. Ultimately, the number of patient and control samples required is an interplay between analytical variation and the number of data points (metabolites) obtained. The patients (or controls) were either (a) fasting overnight and unmedicated/withdrawn from medication [71,86], (b) fasting and on medication [52,66,67,71,72,76], (c) not fasting and unmedicated/withdrawn from medication [55,56,70,87] or (d) not fasting and on medication [51,56,87]. In some studies, only blood, CSF or urine was analyzed, whereas in other studies, both serum and urine or CSF and plasma were analyzed. Combined analyses of different biofluids from the same patients in the same study are more useful, as some changes may be present in CSF, showing disturbances of metabolites that may affect brain function or may be altered because of a localized brain lesion, but not in plasma or urine, which reflect overall body metabolism. Some compounds can easily cross the blood brain barrier and enter the CSF, whereas other compounds do not. On the other hand, most metabolites, including brain-derived metabolites such as HVA or N-acetylaspartate, are more abundant in urine (at least a factor of 10) as compared to blood or CSF [88] and may therefore provide a more reliable metabolic index of a disease process than fluctuating CSF or plasma levels.

In the studies listed in Table 1, different metabolic profiles were detected between controls and PD or subgroups of PD, but many of the compounds separating PD from controls [50,56] or PD-LRRK2 from other PD cases [51] were not structurally identified. Employing metabolomics and pathway enrichment tools, changes were found in purine metabolism [51,86], oxidative stress/redox homeostasis [53,56,66], energy metabolism (glycolysis and TCA cycle) [54,55,72], fatty acid metabolism [54,71,76,86], branched chain amino acids (leucine, isoleucine, valine) [54,72], phenylalanine and tyrosine metabolism [66,67,76,86], tryptophan metabolism [53,54,66,67,76], glycine derivation (related to FA metabolism) and steroidogenesis [67,76]. Correlations with the progression of PD were found for changes in phenylalanine, purine and FA metabolism, serine [86], polyamines [70] and tryptophan metabolism via the kynurenine pathway in urine [67,76], plasma and CSF [52].

Some studies reported no effect of L-DOPA/peripheral decarboxylase inhibitor treatment in metabolic profiling [50], but there are profound metabolic effects of this type of treatment on aromatic amino acid metabolism (tyramine, tryptophan) in plasma, CSF [52] and urine [89,90]. Thus, dopaminergic drug treatments should be taken into account when metabolite profiles are to be compared between PD and controls or between various PD subgroups. If samples are used from PD patients on anti-Parkinsonian medication, including L-DOPA, it is advised to collect the samples within a fixed interval after drug intake. In this way, medication-induced changes in tyrosine metabolism and related pathways can be studied and compared between different PD groups, e.g., with and without L-DOPA-induced dyskinesia [52].

The recent study by LeWitt et al. [86] in unmedicated PD patients addressed the question: How informative is the initial biochemical profile or its change in later collected specimens for predicting disease progression? The authors concluded that CSF-HVA is a poor predictor of PD progression, but that several purines (compounds with xanthine structure) and some medium- or long-chain FA correlated strongly with worsening of Unified Parkinson’s Disease Rating Scale (UPDRS) scores.

### 4.2. Metabolomic Studies in Experimental Models of PD (Table 2)

The advantages and disadvantages of both toxic and genetic animal models of PD have been extensively reviewed by various authors [9,99,100,101,102]. Animal models of PD have been used to study markers of dopaminergic degeneration, changes in receptors or alteration in blood flow or energy consumption using PET or NMR imaging [103]. In vitro models of PD include cell and slice culture models [104] and most recently also differentiated neurons from human iPS cells [105,106]. However, only a few experimental studies used a metabolomic approach to identify novel biomarkers of PD. We have summarized these studies in Table 2.

Table 2 includes one in vitro model, i.e., neuroblastoma cells treated with different toxins [93], the MPTP mouse model [91], Parkin knock-out mice [92], transgenic alpha-syn A53T mice [95], a 6-hydroxydopamine-lesioned rat model [96], a rotenone-treated rat model [97], an MPTP–treated goldfish model [94] and alpha-synuclein overexpressing *Drosophila* [76] or paraquat-treated fruit flies [98]. In all animal studies, metabolite profiles were assessed in brain tissues and in the vitro study in cell extracts and culture medium. Thus, a comparison with human studies where metabolites are usually determined in CSF, plasma or urine is not necessarily feasible.

In Parkin knock-out mice [92], no differences were found in ATP or energy-related metabolites, whereas treatment with the mitochondrial uncoupler CCCP caused a marked drop in ATP. Apparently, in Parkin KO mice, mitochondrial function is not impaired to a degree that causes changes in TCA intermediates or other compounds related to energy metabolism.

In the study using transgenic A53T and wildtype mice [95], not only the effect of the genotype, but also age-related changes were studied in wildtype and transgenic mice. The study reported an interaction between aging and genotype on guanosine levels (purine metabolism), whereas changes in alanine, glutathione and acetyl-CoA metabolism were age-related in both wildtype and Tg mice. A change in purine metabolism (reduced uric acid) was also observed in the paraquat fly model [98]. Changes in energy metabolism were observed in the MPTP mouse [91], neuroblastoma cell cultures treated with toxins [93], MPTP-treated goldfish [94] and paraquat-treated flies [98]. Changes in FA metabolism were reported in different animal models using mitochondrial toxins, including the MPTP mouse [91], MPTP-treated goldfish [94] and rotenone-treated rats [97]. Branched chain amino acid (BCAA) metabolism was altered in the MPTP-treated goldfish [94] (BCAA levels increased) and paraquat-treated flies [98] (BCAA levels decreased). Tyrosine metabolism was altered in the MPTP mouse [91], and tryptophan metabolism (increased KYN/KYNA ratio) was affected in alpha-syn-overexpressing flies [76].

In conclusion, metabolite profiling in brain tissues of different animal models listed in Table 2 revealed changes in some of the pathways identified in the clinical studies of Table 1. However, the potential of using animal studies of PD for longitudinal metabolic profiling in CSF, plasma, urine and brain tissues is not fully explored. In addition, the effects of chronic drug treatments (e.g., L-DOPA) and the relationship between changes in metabolite profiles in brain tissue and various body fluids, CSF, plasma and urine still require further studies.

## 5. General Discussion

Both clinical (Table 1) and experimental studies (Table 2) have consistently shown alterations in alanine metabolism, branched chain amino acids (BCAA) metabolism, FA metabolism and steroidogenesis, all pointing to mitochondrial dysfunction in PD. BCAAs are transported into the brain via the large neutral amino acid transporter [107]. Since aromatic amino acids (tryptophan, phenylalanine, tyrosine, but also L-DOPA) use the same transporter, L-DOPA treatment of PD patients may interfere with BCAA plasma levels. We are not aware of clinical or experimental biomarker studies that have addressed the relationship between chronic L-DOPA use and circulating BCAA levels. An elevation in BCAAs (leucine, isoleucine, valine) has also been associated with mitochondrial respiratory chain disease [108], insulin resistance and the development of type 2 diabetes [109,110]. In Alzheimer’s disease (AD) [111] and in patients with pyruvate dehydrogenase deficiency [108], serum levels of valine are decreased, but in a mouse model of AD BCAA levels, including valine, they were reported to be increased [112]. BCAA plays an important role in bioenergetics, protein synthesis and mitochondrial biogenesis. In addition, BCAA regulates macroautophagy (and mitophagy) by activating mammalian target of rapamycin complex 1 (mTORC1) [113]. Activation of mTORC1 inhibits macroautophagy, whereas inhibition of mTORC1 by rapamycin or other means can provide neuroprotection in animal models of PD [114,115] and attenuate L-DOPA–induced dyskinesia in 6-OHDA lesioned rodents [116,117]. Changes in concentrations or abundance (up and down) of related metabolites can be expressed as ratios. Accordingly, in mitochondrial disease, alterations in the ratios of alanine to glutamate, BCAA to glutamate or lactate to pyruvate have been reported [108]. We propose to look at such ratios at the individual patient level in PD metabolite profiling studies.

An accumulation of free fatty acid in plasma or alterations in brain tissue (up- or down-regulations) has been reported in PD (Table 1) and experimental models of PD (Table 2). Such changes are also observed in disorders associated with mitochondrial dysfunction [118]. Alterations of phosphatidylcholine and lysophosphatidylcholine lipids may not only point to mitochondrial dysfunction and/or neuronal cell loss, but may also play a role in pro-apoptotic or anti-apoptotic signaling [96].

Several metabolomics studies listed in Table 1 and Table 2 identified decreased purine metabolism in PD or models of PD. The end product of purine metabolism, uric acid, is a scavenger of reactive oxygen and reactive nitrogen species and may thus reduce oxidative/nitrative stress. Low uric acid levels have been associated with PD and increased progression of disease in epidemiological studies [26,119]. In contrast, dietary intake of caffeine, a xanthine (purine) compound, has been associated with reduced risk of PD and reduced risk of developing L-DOPA-induced dyskinesia [120,121].

Global metabolite changes in body fluids may reflect a (neuro)pathological process (e.g., mitochondrial dysfunction, inflammation or deficits in autophagy-lysosomal function), but do not localize the damage to specific brain circuits or neuronal cell types. Neurological and psychiatric symptoms (and the diagnosis of a specific disease entity) are highly dependent on which brain regions, neuronal circuitries, cell types or transmitter systems are affected by the disease. Thus, the neurochemical characteristics of the patients should be taken into account in conjunction with more global metabolic disturbances, such as changes in FA metabolism or BCAA.

A myriad of small molecules related to amino acid and/or FA metabolism, such as kynurenine metabolites [122], glycine, serine [123], n-acetylated amino acids or endocannabinoids [124], act as neuromodulators on, e.g., receptor-ion-channel complexes (e.g., NMDA, nicotine ACh receptors), G protein coupled receptors, voltage-gated calcium channels or glycine transporters. Disturbances of the metabolism of these neuromodulators or alterations in the ratios of neuroactive metabolites may contribute to neurological and psychiatric diseases. Alterations in kynurenine (KYN) metabolism, which may give rise to either neurotoxic compounds such as 3-hydrokynurenine (3-HK) or neuroprotective/neuromodulating compounds like kynurenic acid (KYNA), are often expressed as ratios 3-HK/KYNA [52,53] or KYN/KYNA [76] and indicate whether the neurotoxic or neuro-modulating branch of the KYN pathway is favored in the individual patient. Thus, metabolite profiling of body fluids in PD and other brain diseases should also focus on low abundance (nM range) small molecules known to act as neuromodulators, since abnormalities in such compounds can potentially be treated with specific enzyme inhibitors.

## 6. Conclusions and Future Perspectives

**Figure 1:**
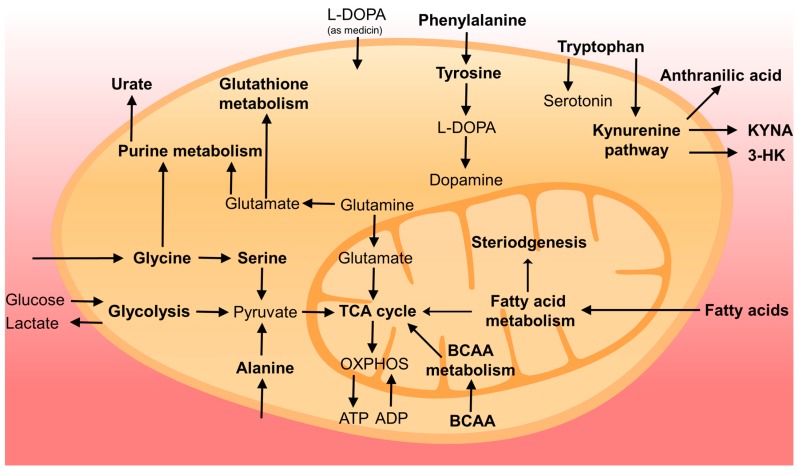
Overview of cellular metabolism changed in PD. Pathways or compounds specifically found in the literature are marked in bold. For simplicity, not all intermediates and reversible processes are shown.

Metabolite profiling of body fluids of PD patients or patients at risk developing PD (e.g., REM sleep disorders) is a powerful tool to identify novel biomarkers for early diagnosis, prognosis and monitoring of disease progression. In Figure 1, we summarized the metabolic pathways and related metabolites showing alterations in PD based on the current literature. However, still much validation work and improvement of procedures are needed before metabolite profiling can be implemented in the clinic. Anti-Parkinson medication, in particular L-DOPA treatment, is a major confounding factor in metabolite profiling studies, and care should be taken to collect biofluid samples at fixed time points after the intake of medication or after drug-wash-out (if this is ethically possible). The identification and quantification of all compounds showing statistical differences between controls and PD patients is still a major challenge. In future studies, it will be important to confirm the identification and to quantify the compounds of interest using isotope-labelled standards (those showing differences between PD and controls) using a targeted approach during data acquisition. Absolute quantification is important in order to compare results with the literature and to check whether similar levels were detected in different control groups. From the point of view of analytical laboratories, it is also mandatory to document procedure variation (inter- and intra-assay), normal ranges and robustness. We recommend also to collect, if possible, samples from different biofluids from the same patients, (e.g., CSF, plasma, saliva and urine) in order to study whether altered metabolite profiles in CSF are reflected in plasma, saliva or urine.

A hot topic in current PD research is the emerging role of the gut flora in the disease process [125]. Case-control studies have shown differences in the composition of gut microbiota between PD patients and age-matched controls [126,127,128], and in addition to changes in certain gut bacteria, one study reported reductions in fecal short chain fatty acids (acetate, propionate, butyrate) [129]. Alterations in the gut microbiome also have effects on drug metabolism, and vice versa, chronic drug treatment may affect the composition of the gut microbiota [130]. In a recent experimental study [131], it was shown that colonization of alpha-synuclein-overexpressing mice with microbiota from PD-affected patients enhances physical impairment compared to mice with microbiota transplants from healthy human donors. Such an approach could also be used to study differences in metabolite profiles in plasma and urine of wildtype or transgenic mice (or rats) transplanted with gut microbiota from PD patients and from healthy controls.

It has been suggested that precision medicine applied to PD should be based on biomarker profiles instead of clinical features or at least on a combination of the clinical features and molecular profiling. Future advanced metabolomics studies in PD and related disorders will be directed toward answering the question of whether differences in metabolic profiles can be used to stratify patients into different therapeutic regimens. This will, however, also require a further understanding of underlying disease mechanisms and the identification of novel drug targets, as well as fully-documented and validated methods.

## Figures and Tables

**Table 1:** Overview of metabolomic studies in PD clinical research. LCECA, liquid chromatography electrochemistry array; LID, L-DOPA-induced-dyskinesia; BCAA, branched chain amino acid.

Article	Analytical Platform	Statistics	Patients (#)	Pathway/Compound	
				Increased in PD	Decreased in PD
Bogdanov et al., 2008 [56]	LCECA	PLS-DA *t*-test	PD (60), controls (25)	B: Glutathione metabolism (GSH)	B: Purine metabolism (uric acid )
Johansen et al., 2009 [51]	LCECA	PLS-DA	PD (41), PD-LRRK2 (12), healthy-LRRK2 (21), controls (25)		B: Purine metabolism (uric acid, hypoxanthine)
Ahmed et al., 2009 [55]	H-NMR	PLS-DA, ANOVA	PD (43), controls (37)	B: Energy metabolism (pyruvate)	B: Energy metabolism (TCA metabolites, creatinine)
Roede et al., 2013 [70]	LC-MS^2 +^	(O)PLS-DA *t*-test	PD rapid progress (39), PD slow progress (41), Controls (20)	B: Polyamine metabolism (N8-acetylspermidine)	
LeWitt et al., 2013 [53]	LC-MS^2 +/−^ GC-MS	*t*-test ROC curve	PD (48), controls (57)	C: Kynurenine metabolism (3-HK/KYNA)	C: Acetylated amino acids, C: glutathione metabolism (GSSG)
Trupp et al., 2014 [54]	GC-MS	(O)PLS-DA	PD (20), controls (20)	B: Amino acids (methionine, threonine, alanine, serine), glutathione metabolism (pyroglutamate), ketoleucine	C: Energy metabolism (creatinine), tryptophan metabolism (tryptophan) B: FA metabolism (C16 and C18)
Öhman and Forsgren 2015 [87]	H-NMR	Multivariate, univariate	PD (10), controls (10)		C: Amino acids (alanine), energy metabolism (creatinine), sugars (mannose)
Luan et al., 2015 [67]	LC-MS ^+/−^	(O)PLS-DA ROC curve	PD (106), controls (104)	U: BCAA, Glycine derivatives, histidine metabolism, tryptophan/kynurenine metabolism, phenylalanine metabolism , purine metabolism, steroidogenesis	
Luan et al., 2015 [76]	LC-MS ^+/−^ GC-MS	(O)PLS-DA	PD (92), controls (65)		
Hatano et al., 2016 [66]	LC-MS ^+/−^ GC-MS	*t*-test	PD (35), controls (15)	U: Phenylalanine metabolism (phenylacetate)	B: Bilirubin/biliverdin, tryptophan metabolism
Wuolikainen et al., 2016 [72]	LC-MS ^+/−^ GC-MS	(O)PLS-DA	PD (22), ALS (22), controls (28)	B and C: Amino acids (alanine) C: BCAA (leucine, isoleucine)	
LeWitt et al., 2017 [86]	LC-MS ^+/−^ GC-MS	Multivariate *t*-test	PD (49); collected twice with an interval of up to 2 years	B: FA metabolism (medium-long chain FA), phenylalanine metabolism (aspartylphenylalanine, benzoate), serine metabolism (serine)	B: Purine metabolism (inosine)
Burté et al., 2017 [71]	LC-MS^2 +/−^ GC-MS	PCA (O)PLS-DA	PD early stage (41), Controls (40)	B: FA metabolism (acylcarnitine), histidine metabolism (1-methylhistamine)	
Havelund et al., 2017 [52]	LC-MS^+^	ANOVA	PD (26), PD-LID (10), controls (14)	B: Kynurenine metabolism (3-HK/KYNA)	B and C: Kynurenine metabolism (anthranilic acid )

^2^ Data-dependent MS/MS; ^+/−^ shows which MS ion mode was used; B/C/U: indicate in which biofluid (B: blood, C: CSF, U: urine) the compound/pathway was found.

**Table 2:** Overview of metabolomic studies in PD model organisms. DOPAC, dihydroxyphenylacetic acid.

Article	Analytical Platform	Statistics	Model (Treatment)	Tissue or Cells	Pathway/Compound	
					Increased in PD	Decreased in PD
Li et al., 2013 [91]	LC-MS ^+^	PCA, PLS-DA, *t*-test	Mice (MPTP-treated mice, *Acanthopanax senticosus* harms)	Midbrain	Ceramide (d18:0/18:0), FA metabolism (lysoPC 20:4), methionine metabolism (5-methylthioadenosine), morphiceptin, sphingolipid metabolism (phytosphingosine-1-P), tetracosanoylglycine, tyrosine metabolism (L-DOPA)	
Poliquin et al., 2013 [92]	LC-MS ^+/−^	Not reported	Parkin KO mice (complex I inhibitor)	Brain slices		Energy metabolism (ATP)
Lei et al., 2014 [93]	H-NMR MS	PLS-DA, ANOVA	Neuroblastoma cells (6-OHDA, MPP^+^, rotenone, or paraquat)	Cells	Energy metabolism (pentose phosphate pathway), sugars (heptose, hexose)	Energy metabolism (TCA cycle), amino acid (glutamate), tyrosine metabolism (dopamine)
Lu et al., 2014 [94]	H-NMR LC-MS ^+/−^	PCA, OPLS-DA, *t*-test, Mann−Whitney U-test	Goldfish (MPTP-treated)	Whole brain	Alanine metabolism (alanine, alanylalanine), amino acids (taurine), BCAA (leucine, isoleucine, valine), energy metabolism (creatinine), FA metabolism (18:2, total FA), sugars (myo-inositol, a glial marker)	Energy metabolism (TCA metabolites), FA metabolism (n-3 FA, unsaturated FA), glutamine metabolism, neuronal injury markers, tyrosine metabolism (dopamine, DOPAC)
Chen et al., 2015 [95]	LC-MS^2 +/−^ GC-MS	PCA, ANOVA, Random forest	Mice (alpha-syn A53T transgenic)	Forebrain and midbrain	Alanine metabolism, acetyl-CoA biosynthesis pathways	Purine metabolism (guanosine)
Farmer et al., 2015 [96]	LC-MS^2 +^	*t*-test	Rats (6-OHDA)	Substantia nigra	Lysophosphatidylcholine (C16:0, 18:1)	Lysophosphatidylcholine, phosphatidylcholine species (12 species)
Tyurina et al., 2015 [97]	LC-MS ^−^		Rats (rotenone)	Substantia nigra (SN) Blood (B)	SN: Mono-oxygenated cardiolipin metabolism, B: Polyunsaturated FA cardiolipin	SN **:** unsaturated FA cardiolipin species
Luan et al., 2015 [67]	LC-MS ^+^	Mann−Whitney U-test	*Drosophila* (alpha-syn overexpressing)	Whole flies	Kynurenine metabolism (kynurenine/KYNA)	
Shukla et al., 2016 [98]	LCECA GC-MS	*t*-test	*Drosophila* (paraquat)	Fly heads	Alanine metabolism (alanine), energy metabolism (lactate acid), FA metabolism (hexadecanoic acid, oleic acid), glycerolipid metabolism, glycine metabolism (glycine), sugars (inositol, myo-inositol)	Amino acids (γ-aminobutyric acid, proline), BCAA (isoleucine, leucine, valine), purine metabolism (uric acid), sugars (glucose, galactose, trehalose), tyrosine metabolism (dopamine)

^2^ Data-dependent MS/MS, ^+/−^ show which MS ion mode was used.

## References

[1] De Lau L.M., Breteler M.M. (2006). Epidemiology of parkinson’s disease. Lancet. Neurol..

[2] Adler C.H., Beach T.G., Hentz J.G., Shill H.A., Caviness J.N., Driver-Dunckley E., Sabbagh M.N., Sue L.I., Jacobson S.A., Belden C.M. (2014). Low clinical diagnostic accuracy of early *vs.* advanced Parkinson disease: Clinicopathologic study. Neurology.

[3] Marek K., Innis R., van Dyck C., Fussell B., Early M., Eberly S., Oakes D., Seibyl J. (2001). [123I] β-CIT SPECT imaging assessment of the rate of Parkinson’s disease progression. Neurology.

[4] Morrish P.K., Sawle G.V., Brooks D.J. (1995). Clinical and [18F] dopa PET findings in early Parkinson’s disease. J. Neurol. Neurosurg. Psychiatry.

[5] Fearnley J.M., Lees A.J. (1991). Ageing and Parkinson’s disease: Substantia nigra regional selectivity. Brain.

[6] Berg D., Postuma R.B., Adler C.H., Bloem B.R., Chan P., Dubois B., Gasser T., Goetz C.G., Halliday G., Joseph L. (2015). MDS research criteria for prodromal Parkinson’s disease. Mov. Disord..

[7] Schapira A.H.V., Chaudhuri K.R., Jenner P. (2017). Non-motor features of Parkinson disease. Nat. Rev. Neurosci..

[8] Ory-Magne F., Corvol J.C., Azulay J.P., Bonnet A.M., Brefel-Courbon C., Damier P., Dellapina E., Destee A., Durif F., Galitzky M. (2014). Withdrawing amantadine in dyskinetic patients with Parkinson disease: The amandysk trial. Neurology.

[9] Bastide M.F., Meissner W.G., Picconi B., Fasano S., Fernagut P.O., Feyder M., Francardo V., Alcacer C., Ding Y., Brambilla R. (2015). Pathophysiology of L-DOPA-induced motor and non-motor complications in Parkinson’s disease. Prog. Neurobiol..

[10] Franco-Iborra S., Vila M., Perier C. (2016). The Parkinson disease mitochondrial hypothesis: Where are we at?. Neuroscientist.

[11] Truban D., Hou X., Caulfield T.R., Fiesel F.C., Springer W. (2017). PINK1, parkin, and mitochondrial quality control: What can we learn about Parkinson’s disease pathobiology?. J. Parkinsons Dis..

[12] Schapira A.H., Chiasserini D., Beccari T., Parnetti L. (2016). Glucocerebrosidase in Parkinson’s disease: Insights into pathogenesis and prospects for treatment. Mov. Disord..

[13] Moors T., Paciotti S., Chiasserini D., Calabresi P., Parnetti L., Beccari T., van de Berg W.D. (2016). Lysosomal dysfunction and alpha-synuclein aggregation in Parkinson’s disease: Diagnostic links. Mov. Disord..

[14] Hong Z., Shi M., Chung K.A., Quinn J.F., Peskind E.R., Galasko D., Jankovic J., Zabetian C.P., Leverenz J.B., Baird G. (2010). DJ-1 and alpha-synuclein in human cerebrospinal fluid as biomarkers of Parkinson’s disease. Brain.

[15] Shi M., Furay A.R., Sossi V., Aasly J.O., Armaly J., Wang Y., Wszolek Z.K., Uitti R.J., Hasegawa K., Yokoyama T. (2012). DJ-1 and alphaSYN in LRRK2 CSF do not correlate with striatal dopaminergic function. Neurobiol. Aging.

[16] Parnetti L., Castrioto A., Chiasserini D., Persichetti E., Tambasco N., El-Agnaf O., Calabresi P. (2013). Cerebrospinal fluid biomarkers in Parkinson disease. Nat. Rev. Neurol..

[17] Parnetti L., Chiasserini D., Persichetti E., Eusebi P., Varghese S., Qureshi M.M., Dardis A., Deganuto M., De Carlo C., Castrioto A. (2014). Cerebrospinal fluid lysosomal enzymes and alpha-synuclein in Parkinson’s disease. Mov. Disord..

[18] Parnetti L., Cicognola C., Eusebi P., Chiasserini D. (2016). Value of cerebrospinal fluid alpha-synuclein species as biomarker in Parkinson’s diagnosis and prognosis. Biomark. Med..

[19] Matrone C., Dzamko N., Madsen P., Nyegaard M., Pohlmann R., Sondergaard R.V., Lassen L.B., Andresen T.L., Halliday G.M., Jensen P.H. (2016). Mannose 6-phosphate receptor is reduced in -synuclein overexpressing models of Parkinsons disease. PLoS ONE.

[20] Van Dijk K.D., Persichetti E., Chiasserini D., Eusebi P., Beccari T., Calabresi P., Berendse H.W., Parnetti L., van de Berg W.D. (2013). Changes in endolysosomal enzyme activities in cerebrospinal fluid of patients with Parkinson’s disease. Mov. Disord..

[21] Kang J.H., Mollenhauer B., Coffey C.S., Toledo J.B., Weintraub D., Galasko D.R., Irwin D.J., Van Deerlin V., Chen-Plotkin A.S., Caspell-Garcia C. (2016). CSF biomarkers associated with disease heterogeneity in early Parkinson’s disease: The Parkinson’s progression markers initiative study. Acta Neuropathol..

[22] Hall S., Surova Y., Ohrfelt A., Swedish Bio F.S., Blennow K., Zetterberg H., Hansson O. (2016). Longitudinal measurements of cerebrospinal fluid biomarkers in Parkinson’s disease. Mov. Disord..

[23] Schrag A., Siddiqui U.F., Anastasiou Z., Weintraub D., Schott J.M. (2017). Clinical variables and biomarkers in prediction of cognitive impairment in patients with newly diagnosed Parkinson’s disease: A cohort study. Lancet Neurol..

[24] Doppler K., Jentschke H.M., Schulmeyer L., Vadasz D., Janzen A., Luster M., Hoffken H., Mayer G., Brumberg J., Booij J. (2017). Dermal phospho-alpha-synuclein deposits confirm REM sleep behaviour disorder as prodromal Parkinson’s disease. Acta Neuropathol..

[25] Espay A.J., Schwarzschild M.A., Tanner C.M., Fernandez H.H., Simon D.K., Leverenz J.B., Merola A., Chen-Plotkin A., Brundin P., Kauffman M.A. (2017). Biomarker-driven phenotyping in Parkinson’s disease: A translational missing link in disease-modifying clinical trials. Mov. Disord..

[26] Ascherio A., LeWitt P.A., Xu K., Eberly S., Watts A., Matson W.R., Marras C., Kieburtz K., Rudolph A., Bogdanov M.B. (2009). Urate as a predictor of the rate of clinical decline in Parkinson disease. Arch Neurol..

[27] LeWitt P., Schultz L., Auinger P., Lu M., Parkinson Study Group, Datatop Investigators (2011). CSF xanthine, homovanillic acid, and their ratio as biomarkers of Parkinson’s disease. Brain Res..

[28] Mischley L.K., Standish L.J., Weiss N.S., Padowski J.M., Kavanagh T.J., White C.C., Rosenfeld M.E. (2016). Glutathione as a biomarker in Parkinson’s disease: Associations with aging and disease severity. Oxid. Med. Cell Longev..

[29] LeWitt P.A., Galloway M.P., Matson W., Milbury P., McDermott M., Srivastava D.K., Oakes D. (1992). Markers of dopamine metabolism in Parkinson’s disease. Neurology.

[30] Goldstein D.S., Holmes C., Sharabi Y. (2012). Cerebrospinal fluid biomarkers of central catecholamine deficiency in Parkinson’s disease and other synucleinopathies. Brain.

[31] Andersen A.D., Blaabjerg M., Binzer M., Kamal A., Thagesen H., Kjaer T.W., Stenager E., Gramsbergen J.B.P. (2017). Cerebrospinal fluid levels of catecholamines and its metabolites in Parkinson’s disease: Effect of L-DOPA treatment and changes in levodopa-induced dyskinesia. J. Neurochem..

[32] Goldstein D.S. (2013). Biomarkers, mechanisms, and potential prevention of catecholamine neuron loss in Parkinson disease. Adv. Pharmacol..

[33] Andersen A.D., Binzer M., Stenager E., Gramsbergen J.B. (2017). Cerebrospinal fluid biomarkers for Parkinson’s disease—A systematic review. Acta Neurol. Scand..

[34] Jimenez-Jimenez F.J., Alonso-Navarro H., Garcia-Martin E., Agundez J.A. (2014). Cerebrospinal fluid biochemical studies in patients with Parkinson’s disease: Toward a potential search for biomarkers for this disease. Front. Cell Neurosci..

[35] Molina J.A., Jimenez-Jimenez F.J., Gomez P., Vargas C., Navarro J.A., Orti-Pareja M., Gasalla T., Benito-Leon J., Bermejo F., Arenas J. (1997). Decreased cerebrospinal fluid levels of neutral and basic amino acids in patients with Parkinson’s disease. J. Neurol. Sci..

[36] Bowen B.C., Block R.E., Sanchez-Ramos J., Pattany P.M., Lampman D.A., Murdoch J.B., Quencer R.M. (1995). Proton MR spectroscopy of the brain in 14 patients with Parkinson disease. AJNR Am. J. Neuroradiol..

[37] Henchcliffe C., Shungu D.C., Mao X., Huang C., Nirenberg M.J., Jenkins B.G., Beal M.F. (2008). Multinuclear magnetic resonance spectroscopy for *in vivo* assessment of mitochondrial dysfunction in Parkinson’s disease. Ann. N. Y. Acad. Sci..

[38] Ciurleo R., Di Lorenzo G., Bramanti P., Marino S. (2014). Magnetic resonance spectroscopy: An in vivo molecular imaging biomarker for Parkinson’s disease?. Biomed. Res. Int..

[39] Levin B.E., Katzen H.L., Maudsley A., Post J., Myerson C., Govind V., Nahab F., Scanlon B., Mittel A. (2014). Whole-brain proton MR spectroscopic imaging in Parkinson’s disease. J. Neuroimaging.

[40] Seraji-Bozorgzad N., Bao F., George E., Krstevska S., Gorden V., Chorostecki J., Santiago C., Zak I., Caon C., Khan O. (2015). Longitudinal study of the substantia nigra in Parkinson disease: A high-field ^1^H-MR spectroscopy imaging study. Mov. Disord..

[41] Weiduschat N., Mao X., Beal M.F., Nirenberg M.J., Shungu D.C., Henchcliffe C. (2015). Usefulness of proton and phosphorus MR spectroscopic imaging for early diagnosis of Parkinson’s disease. J. Neuroimaging.

[42] Lloyd S.M., Arnold J., Sreekumar A. (2015). Metabolomic profiling of hormone-dependent cancers: A bird’s eye view. Trends Endocrinol. Metab..

[43] Lei S., Powers R. (2013). NMR metabolomics analysis of Parkinson’s disease. Curr. Metabolomics.

[44] Jove M., Portero-Otin M., Naudi A., Ferrer I., Pamplona R. (2014). Metabolomics of human brain aging and age-related neurodegenerative diseases. J Neuropathol. Exp. Neurol..

[45] Ibanez C., Cifuentes A., Simo C. (2015). Recent advances and applications of metabolomics to investigate neurodegenerative diseases. Int. Rev. Neurobiol..

[46] Kori M., Aydin B., Unal S., Arga K.Y., Kazan D. (2016). Metabolic biomarkers and neurodegeneration: A pathway enrichment analysis of Alzheimer’s disease, Parkinson’s disease, and amyotrophic lateral sclerosis. Omics.

[47] Fiehn O. (2002). Metabolomics-the link between genotypes and phenotypes. Plant Mol. Biol..

[48] Villas-Boas S.G., Mas S., Akesson M., Smedsgaard J., Nielsen J. (2005). Mass spectrometry in metabolome analysis. Mass Spectrom. Rev..

[49] Wishart D.S. (2016). Emerging applications of metabolomics in drug discovery and precision medicine. Nat. Rev. Drug Discov..

[50] Michell A.W., Mosedale D., Grainger D.J., Barker R.A. (2008). Metabolomic analysis of urine and serum in parkinson’s disease. Metabolomics.

[51] Johansen K.K., Wang L., Aasly J.O., White L.R., Matson W.R., Henchcliffe C., Beal M.F., Bogdanov M. (2009). Metabolomic profiling in LRRK2-related parkinson’s disease. PLoS ONE.

[52] Havelund J.F., Andersen A.D., Binzer M., Blaabjerg M., Heegaard N.H.H., Stenager E., Faergeman N.J., Gramsbergen J.B. Changes in Kynurenine Pathway Metabolism in Parkinson Patients with L-DOPA-Induced Dyskinesia. http://onlinelibrary.wiley.com/doi/10.1111/jnc.14104/full.

[53] Lewitt P.A., Li J., Lu M., Beach T.G., Adler C.H., Guo L., The Arizona Parkinson’s Disease Consortium (2013). 3-hydroxykynurenine and other Parkinson’s disease biomarkers discovered by metabolomic analysis. Mov. Disord..

[54] Trupp M., Jonsson P., Ohrfelt A., Zetterberg H., Obudulu O., Malm L., Wuolikainen A., Linder J., Moritz T., Blennow K. (2014). Metabolite and peptide levels in plasma and CSF differentiating healthy controls from patients with newly diagnosed Parkinson’s disease. J. Parkinsons Dis..

[55] Ahmed S.S., Santosh W., Kumar S., Christlet H.T. (2009). Metabolic profiling of Parkinson’s disease: Evidence of biomarker from gene expression analysis and rapid neural network detection. J. Biomed. Sci..

[56] Bogdanov M., Matson W.R., Wang L., Matson T., Saunders-Pullman R., Bressman S.S., Flint Beal M. (2008). Metabolomic profiling to develop blood biomarkers for Parkinson’s disease. Brain.

[57] Trushina E., Mielke M.M. (2014). Recent advances in the application of metabolomics to Alzheimer’s disease. Biochim. Biophys. Acta.

[58] Kaddurah-Daouk R., Kristal B.S., Weinshilboum R.M. (2008). Metabolomics: A global biochemical approach to drug response and disease. Annu. Rev. Pharmacol. Toxicol..

[59] Kristal B.S., Vigneau-Callahan K., Matson W.R. (2002). Simultaneous analysis of multiple redox-active metabolites from biological matrices. Methods Mol. Biol..

[60] Shi H., Vigneau-Callahan K.E., Matson W.R., Kristal B.S. (2002). Attention to relative response across sequential electrodes improves quantitation of coulometric array. Anal. Biochem..

[61] Marion D. (2013). An introduction to biological NMR spectroscopy. Mol. Cell Proteom..

[62] De Hoffmann E., Stroobant V. (2007). Mass Spectrometry: Principles and Applications.

[63] Dias D.A., Jones O.A., Beale D.J., Boughton B.A., Benheim D., Kouremenos K.A., Wolfender J.L., Wishart D.S. (2016). Current and future perspectives on the structural identification of small molecules in biological systems. Metabolites.

[64] Dunand M., Gubian D., Stauffer M., Abid K., Grouzmann E. (2013). High-throughput and sensitive quantitation of plasma catecholamines by ultraperformance liquid chromatography-tandem mass spectrometry using a solid phase microwell extraction plate. Anal. Chem..

[65] Barganska Z., Namiesnik J. (2010). Pesticide analysis of bee and bee product samples. Crit. Rev. Anal. Chem..

[66] Hatano T., Saiki S., Okuzumi A., Mohney R.P., Hattori N. (2016). Identification of novel biomarkers for Parkinson’s disease by metabolomic technologies. J. Neurol. Neurosurg. Psychiatry.

[67] Luan H., Liu L.F., Meng N., Tang Z., Chua K.K., Chen L.L., Song J.X., Mok V.C., Xie L.X., Li M. (2015). LC-MS-based urinary metabolite signatures in idiopathic Parkinson’s disease. J. Proteome Res..

[68] Salek R.M., Neumann S., Schober D., Hummel J., Billiau K., Kopka J., Correa E., Reijmers T., Rosato A., Tenori L. (2015). Coordination of standards in metabolomics (COSMOS): Facilitating integrated metabolomics data access. Metabolomics.

[69] Sumner L.W., Amberg A., Barrett D., Beale M.H., Beger R., Daykin C.A., Fan T.W., Fiehn O., Goodacre R., Griffin J.L. (2007). Proposed minimum reporting standards for chemical analysis chemical analysis working group (CAWG) metabolomics standards initiative (MSI). Metabolomics.

[70] Roede J.R., Uppal K., Park Y., Lee K., Tran V., Walker D., Strobel F.H., Rhodes S.L., Ritz B., Jones D.P. (2013). Serum metabolomics of slow *vs.* Rapid motor progression Parkinson’s disease: A pilot study. PLoS ONE.

[71] Burte F., Houghton D., Lowes H., Pyle A., Nesbitt S., Yarnall A., Yu-Wai-Man P., Burn D.J., Santibanez-Koref M., Hudson G. Metabolic Profiling of Parkinson’s Disease and Mild Cognitive Impairment. http://onlinelibrary.wiley.com/doi/10.1002/mds.26992/full.

[72] Wuolikainen A., Jonsson P., Ahnlund M., Antti H., Marklund S.L., Moritz T., Forsgren L., Andersen P.M., Trupp M. (2016). Multi-platform mass spectrometry analysis of the CSF and plasma metabolomes of rigorously matched amyotrophic lateral sclerosis, Parkinson’s disease and control subjects. Mol. Biosyst..

[73] Evans A.M., DeHaven C.D., Barrett T., Mitchell M., Milgram E. (2009). Integrated, nontargeted ultrahigh performance liquid chromatography/electrospray ionization tandem mass spectrometry platform for the identification and relative quantification of the small-molecule complement of biological systems. Anal. Chem..

[74] Markley J.L., Bruschweiler R., Edison A.S., Eghbalnia H.R., Powers R., Raftery D., Wishart D.S. (2017). The future of NMR-based metabolomics. Curr. Opin. Biotechnol..

[75] Lei Z., Huhman D.V., Sumner L.W. (2011). Mass spectrometry strategies in metabolomics. J. Biol. Chem..

[76] Luan H., Liu L.F., Tang Z., Zhang M., Chua K.K., Song J.X., Mok V.C., Li M., Cai Z. (2015). Comprehensive urinary metabolomic profiling and identification of potential noninvasive marker for idiopathic Parkinson’s disease. Sci. Rep..

[77] Patti G.J., Yanes O., Siuzdak G. (2012). Innovation: Metabolomics: The apogee of the omics trilogy. Nat. Rev. Mol. Cell Biol..

[78] Worley B., Powers R. (2013). Multivariate analysis in metabolomics. Curr. Metab..

[79] Eriksson L., Trygg J., Wold S. (2008). CV-ANOVA for significance testing of PLS and OPLS models. J. Chemometr..

[80] Xia J.G., Broadhurst D.I., Wilson M., Wishart D.S. (2013). Translational biomarker discovery in clinical metabolomics: An introductory tutorial. Metabolomics.

[81] Zweig M.H., Campbell G. (1993). Receiver operating characteristic (ROC) plots—A fundamental evaluation tool in clinical medicine. Clin. Chem..

[82] Pepe M.S., Etzioni R., Feng Z., Potter J.D., Thompson M.L., Thornquist M., Winget M., Yasui Y. (2001). Phases of biomarker development for early detection of cancer. J. Natl. Cancer Inst..

[83] Obuchowski N.A., Lieber M.L., Wians F.H. (2004). ROC curves in clinical chemistry: Uses, misuses, and possible solutions. Clin. Chem..

[84] Søreide K. (2009). Receiver-operating characteristic curve analysis in diagnostic, prognostic and predictive biomarker research. J. Clin. Pathol..

[85] Nyamundanda G., Gormley I.C., Fan Y., Gallagher W.M., Brennan L. (2013). Metsizer: Selecting the optimal sample size for metabolomic studies using an analysis based approach. Bioinformatics.

[86] LeWitt P.A., Li J., Lu M., Guo L., Auinger P., Parkinson Study Group, Datatop Investigators (2017). Metabolomic biomarkers as strong correlates of Parkinson disease progression. Neurology.

[87] Ohman A., Forsgren L. (2015). NMR metabonomics of cerebrospinal fluid distinguishes between Parkinson’s disease and controls. Neurosci. Lett..

[88] Wishart D.S., Tzur D., Knox C., Eisner R., Guo A.C., Young N., Cheng D., Jewell K., Arndt D., Sawhney S. (2007). HMDB: The human metabolome database. Nucleic Acids Res..

[89] Davidson D.F., Grosset K., Grosset D. (2007). Parkinson’s disease: The effect of l-dopa therapy on urinary free catecholamines and metabolites. Ann. Clin. Biochem..

[90] Eisenhofer G., Brown S., Peitzsch M., Pelzel D., Lattke P., Glockner S., Stell A., Prejbisz A., Fassnacht M., Beuschlein F. (2014). Levodopa therapy in Parkinson’s disease: Influence on liquid chromatographic tandem mass spectrometric-based measurements of plasma and urinary normetanephrine, metanephrine and methoxytyramine. Ann. Clin. Biochem..

[91] Li X.Z., Zhang S.N., Lu F., Liu C.F., Wang Y., Bai Y., Wang N., Liu S.M. (2013). Cerebral metabonomics study on Parkinson’s disease mice treated with extract of acanthopanax senticosus harms. Phytomedicine.

[92] Poliquin P.O., Chen J., Cloutier M., Trudeau L.E., Jolicoeur M. (2013). Metabolomics and in-silico analysis reveal critical energy deregulations in animal models of Parkinson’s disease. PLoS ONE.

[93] Lei S., Zavala-Flores L., Garcia-Garcia A., Nandakumar R., Huang Y., Madayiputhiya N., Stanton R.C., Dodds E.D., Powers R., Franco R. (2014). Alterations in energy/redox metabolism induced by mitochondrial and environmental toxins: A specific role for glucose-6-phosphate-dehydrogenase and the pentose phosphate pathway in paraquat toxicity. ACS Chem. Biol..

[94] Lu Z., Wang J., Li M., Liu Q., Wei D., Yang M., Kong L. (2014). ^1^H-NMR based metabolomics study on a goldfish model of Parkinson’s disease induced by 1-methyl-4-phenyl-1,2,3,6-tetrahydropyridine (MPTP). Chem. Biol. Interact..

[95] Chen X., Xie C., Sun L., Ding J., Cai H. (2015). Longitudinal metabolomics profiling of Parkinson’s disease-related alpha-synuclein a53t transgenic mice. PLoS ONE.

[96] Farmer K., Smith C.A., Hayley S., Smith J. (2015). Major alterations of phosphatidylcholine and lysophosphotidylcholine lipids in the substantia nigra using an early stage model of Parkinson’s disease. Int. J. Mol. Sci..

[97] Tyurina Y.Y., Polimova A.M., Maciel E., Tyurin V.A., Kapralova V.I., Winnica D.E., Vikulina A.S., Domingues M.R., McCoy J., Sanders L.H. (2015). LC/MS analysis of cardiolipins in substantia nigra and plasma of rotenone-treated rats: Implication for mitochondrial dysfunction in Parkinson’s disease. Free Radic. Res..

[98] Shukla A.K., Ratnasekhar C., Pragya P., Chaouhan H.S., Patel D.K., Chowdhuri D.K., Mudiam M.K. (2016). Metabolomic analysis provides insights on paraquat-induced Parkinson-like symptoms in drosophila melanogaster. Mol. Neurobiol..

[99] Jenner P. (2008). Functional models of Parkinson’s disease: A valuable tool in the development of novel therapies. Ann. Neurol..

[100] Chesselet M.F., Fleming S., Mortazavi F., Meurers B. (2008). Strengths and limitations of genetic mouse models of Parkinson’s disease. Parkinsonism Relat. Disord..

[101] Dehay B., Vila M., Bezard E., Brundin P., Kordower J.H. (2016). Alpha-synuclein propagation: New insights from animal models. Mov. Disord..

[102] Blesa J., Przedborski S. (2014). Parkinson’s disease: Animal models and dopaminergic cell vulnerability. Front. Neuroanat..

[103] Bannon D., Landau A.M., Doudet D.J. How Relevant Are Imaging Findings in Animal Models of Movement Disorders to Human Disease?. https://link.springer.com/article/10.1007/s11910-015-0571-z.

[104] Larsen T.R., Soderling A.S., Caidahl K., Roepstorff P., Gramsbergen J.B. (2008). Nitration of soluble proteins in organotypic culture models of Parkinson’s disease. Neurochem. Int..

[105] Playne R., Connor B. (2017). Understanding Parkinson’s disease through the use of cell reprogramming. Stem cell. Rev..

[106] Torrent R., De Angelis Rigotti F., Dell’Era P., Memo M., Raya A., Consiglio A. (2015). Using ips cells toward the understanding of Parkinson’s disease. J. Clin. Med..

[107] Fernstrom J.D. (2013). Large neutral amino acids: Dietary effects on brain neurochemistry and function. Amino Acids.

[108] Clarke C., Xiao R., Place E., Zhang Z., Sondheimer N., Bennett M., Yudkoff M., Falk M.J. (2013). Mitochondrial respiratory chain disease discrimination by retrospective cohort analysis of blood metabolites. Mol. Genet. Metab..

[109] Connelly M.A., Wolak-Dinsmore J., Dullaart R.P.F. (2017). Branched chain amino acids are associated with insulin resistance independent of leptin and adiponectin in subjects with varying degrees of glucose tolerance. Metab. Syndr. Relat. Disord..

[110] Wang T.J., Larson M.G., Vasan R.S., Cheng S., Rhee E.P., McCabe E., Lewis G.D., Fox C.S., Jacques P.F., Fernandez C. (2011). Metabolite profiles and the risk of developing diabetes. Nat. Med..

[111] Toledo J.B., Arnold M., Kastenmuller G., Chang R., Baillie R.A., Han X., Thambisetty M., Tenenbaum J.D., Suhre K., Thompson J.W. Metabolic Network Failures in Alzheimer’s Disease—A Biochemical Road Map. http://www.sciencedirect.com/science/article/pii/S1552526017300468.

[112] Ruiz H.H., Chi T., Shin A.C., Lindtner C., Hsieh W., Ehrlich M., Gandy S., Buettner C. (2016). Increased susceptibility to metabolic dysregulation in a mouse model of Alzheimer’s disease is associated with impaired hypothalamic insulin signaling and elevated bcaa levels. Alzheimer’s Dement..

[113] Valerio A., D’Antona G., Nisoli E. (2011). Branched-chain amino acids, mitochondrial biogenesis, and healthspan: An evolutionary perspective. Aging.

[114] Bove J., Martinez-Vicente M., Vila M. (2011). Fighting neurodegeneration with rapamycin: Mechanistic insights. Nat. Rev. Neurosci..

[115] Malagelada C., Jin Z.H., Jackson-Lewis V., Przedborski S., Greene L.A. (2010). Rapamycin protects against neuron death in in vitro and in vivo models of Parkinson’s disease. J. Neurosci..

[116] Santini E., Feyder M., Gangarossa G., Bateup H.S., Greengard P., Fisone G. (2012). Dopamine- and cAMP-regulated phosphoprotein of 32-kDa (DARPP-32)-dependent activation of extracellular signal-regulated kinase (ERK) and mammalian target of rapamycin complex 1 (mtorc1) signaling in experimental Parkinsonism. J. Biol. Chem..

[117] Decressac M., Bjorklund A. (2013). mTOR inhibition alleviates L-DOPA-induced dyskinesia in Parkinsonian rats. J. Parkinsons Dis..

[118] Hafizi Abu Bakar M., Kian Kai C., Wan Hassan W.N., Sarmidi M.R., Yaakob H., Zaman Huri H. (2015). Mitochondrial dysfunction as a central event for mechanisms underlying insulin resistance: The roles of long chain fatty acids. Diabetes Metab. Res. Rev..

[119] Schlesinger I., Schlesinger N. (2008). Uric acid in Parkinson’s disease. Mov. Disord..

[120] Wills A.M., Eberly S., Tennis M., Lang A.E., Messing S., Togasaki D., Tanner C.M., Kamp C., Chen J.F., Oakes D. (2013). Caffeine consumption and risk of dyskinesia in CALM-PD. Mov. Disord..

[121] Palacios N., Gao X., McCullough M.L., Schwarzschild M.A., Shah R., Gapstur S., Ascherio A. (2012). Caffeine and risk of Parkinson’s disease in a large cohort of men and women. Mov. Disord..

[122] Schwarcz R., Bruno J.P., Muchowski P.J., Wu H.Q. (2012). Kynurenines in the mammalian brain: When physiology meets pathology. Nat. Rev. Neurosci..

[123] Balu D.T., Coyle J.T. (2015). The NMDA receptor ‘glycine modulatory site’ in schizophrenia: D-serine, glycine, and beyond. Curr. Opin. Pharmacol..

[124] Connor M., Vaughan C.W., Vandenberg R.J. (2010). *N*-acyl amino acids and *N*-acyl neurotransmitter conjugates: Neuromodulators and probes for new drug targets. Br. J. Pharmacol..

[125] Scheperjans F. (2016). Gut microbiota, 1013 new pieces in the Parkinson’s disease puzzle. Curr. Opin. Neurol..

[126] Scheperjans F., Aho V., Pereira P.A., Koskinen K., Paulin L., Pekkonen E., Haapaniemi E., Kaakkola S., Eerola-Rautio J., Pohja M. (2015). Gut microbiota are related to Parkinson’s disease and clinical phenotype. Mov. Disord..

[127] Keshavarzian A., Green S.J., Engen P.A., Voigt R.M., Naqib A., Forsyth C.B., Mutlu E., Shannon K.M. (2015). Colonic bacterial composition in Parkinson’s disease. Mov. Disord..

[128] Hasegawa S., Goto S., Tsuji H., Okuno T., Asahara T., Nomoto K., Shibata A., Fujisawa Y., Minato T., Okamoto A. (2015). Intestinal dysbiosis and lowered serum lipopolysaccharide-binding protein in Parkinson’s disease. PLoS ONE.

[129] Unger M.M., Spiegel J., Dillmann K.U., Grundmann D., Philippeit H., Burmann J., Fassbender K., Schwiertz A., Schafer K.H. (2016). Short chain fatty acids and gut microbiota differ between patients with Parkinson’s disease and age-matched controls. Parkinsonism Relat. Disord..

[130] Wilson I.D., Nicholson J.K. (2017). Gut microbiome interactions with drug metabolism, efficacy, and toxicity. Transl. Res..

[131] Sampson T.R., Debelius J.W., Thron T., Janssen S., Shastri G.G., Ilhan Z.E., Challis C., Schretter C.E., Rocha S., Gradinaru V. (2016). Gut microbiota regulate motor deficits and neuroinflammation in a model of Parkinson’s disease. Cell.

